# Streptococcus suis Serotype 2 Infection Induces Splenomegaly with Splenocyte Apoptosis

**DOI:** 10.1128/spectrum.03210-22

**Published:** 2022-10-26

**Authors:** Shujie Wang, Gang Wang, Yan-Dong Tang, Siqi Li, Lei Qin, Menghang Wang, Yong-Bo Yang, Marcelo Gottschalk, Xuehui Cai

**Affiliations:** a State Key Laboratory of Veterinary Biotechnology, Harbin Veterinary Research Institute, Chinese Academy of Agricultural Sciences, Harbin, China; b Heilongjiang Provincial Key Laboratory of Veterinary Immunology, Harbin, China; c Shandong Provincial Key Laboratory of Animal Biotechnology and Disease Control and Prevention, College of Veterinary Medicine, Shandong Agricultural University, Taian, China; d Research Group on Infectious Diseases in Production Animals (GREMIP) and Swine and Poultry Infectious Diseases Research Center (CRIPA), Faculty of Veterinary Medicine, University of Montreal, Saint-Hyacinthe, Quebec, Canada; Yale University

**Keywords:** *Streptococcus suis*, splenomegaly, apoptosis, pyroptosis, inflammation

## Abstract

Little is known about the damage to the important peripheral immune organ spleen caused by Streptococcus suis infection. In this study, we found that S. suis induced splenomegaly and lymphocyte disruption in spleens of mice. To explore the mechanism of splenic lesions induced by S. suis, we conducted further studies. The results showed that S. suis induced apoptosis in B cells, which is related to the cleavage of caspase-3 and caspase-8, but not the release of apoptosis-inducing factor (AIF). Thus, S. suis induced apoptosis in the spleen through caspase-dependent and AIF-independent pathways. Inflammation lesions induced in the spleen of infected mice were also investigated; we found macrophages increased in histopathological lesions of infected spleens from 12 h postinoculation to 7 days postinoculation (dpi), and the type of increased macrophages was M1 type by confocal microscopy, which can secrete proinflammatory cytokines. Meanwhile, inflammasome NLRP3 and caspase-1 were activated, and gasdermin D (GSDMD) was cleaved, which causes pyroptosis that may result in the release of numerous proinflammatory cytokines. What’s more, the increase of p-JNK and p-p38 indicated that the MAPK pathway was also involved in the proinflammatory responses during S. suis infection, whereas anti-inflammatory responses in spleen were suppressed, with regulatory T cells (Tregs) upregulating at 1 dpi. Taken together, proinflammatory immune responses dominate in early infection, which induce splenomegaly and splenocyte apoptosis. This is the first report of mechanisms associated with S. suis-induced splenic lesions.

**IMPORTANCE** Streptococcus suis serotype 2 is considered an emerging pathogen and represents a threat to humans and animals. The spleen is an important peripheral immune organ, and splenomegaly is a consequence of lesions and an important clinical indicator of S. suis infection. However, knowledge of the mechanisms underlying spleen lesions is still very limited. In the present work, we made the investigation to explain the phenomenon and the related immunomodulation in a mouse infection model. The obtained results show that inflammation contributes to splenomegaly, while apoptosis contributes to lymphocyte disruption in spleens. Related signaling pathways were discovered which have never been associated with S. suis-induced splenic injury. The new knowledge generated will help us better understand the mechanism of S. suis pathogenesis.

## INTRODUCTION

Streptococcus suis is a swine pathogen causing a wide range of infections such as meningitis, septicemia, arthritis, and endocarditis (1, 2). In humans, especially among abattoir workers and swine and pork handlers (3), S. suis type 2 can cause streptococcal toxic shock syndrome (4, 5), which is characterized by acute high fever, vascular collapse, hypotension, shock, and multiple organ failure and can ultimately result in death (4, 6). Thus, S. suis is considered an emerging pathogen and represents a threat to human health.

It has been reported that infection by S. suis may damage central immune organs causing atrophy of the thymus and apoptosis of thymocytes (7). However, little is known so far about the damage to the peripheral immune organs caused by this infection. Peripheral immune organs are the place where immune cells, such as mature T and B lymphocytes, settle and immune response occurs (8). It has been reported that CD4-positive (CD4^+^) T cells in the spleen were significantly reduced after S. suis infection, and the lack of significant expansion of memory T cells compromises the optimal development of adaptive immune responses, including antibody production (9). However, there has been no report of spleen injury and mechanisms caused by S. suis infection.

The spleen is an important peripheral immune organ, which is not only an organ for making white blood cells and filtering and storing blood but also an important base for participating in the immune response (10). Some pathogens, including Mycoplasma gallisepticum, Pneumocystis, Listeria monocytogenes, porcine circovirus type 2, and classical swine fever virus, can cause lymphoid depletion and showed lymphocyte apoptosis in the spleen tissue (11–15). The precise underlying mechanisms vary in different diseases.

Here, C57BL/6 mice were infected by S. suis, which led to spleen swelling with apoptosis in splenic lesions. We aimed to identify the impact and mechanisms of S. suis splenomegaly and splenocyte apoptosis in mice. Our results showed that S. suis triggered caspase-dependent apoptosis in B cells and pyroptosis in macrophages, accompanied by activation of M1 macrophages and the NLRP3 inflammasome, as well as the inhibition of secretion anti-inflammatory cytokines.

## RESULTS

### S. suis infection induces pathological lesions in spleens of C57BL/6 mice.

**FIG 1:**
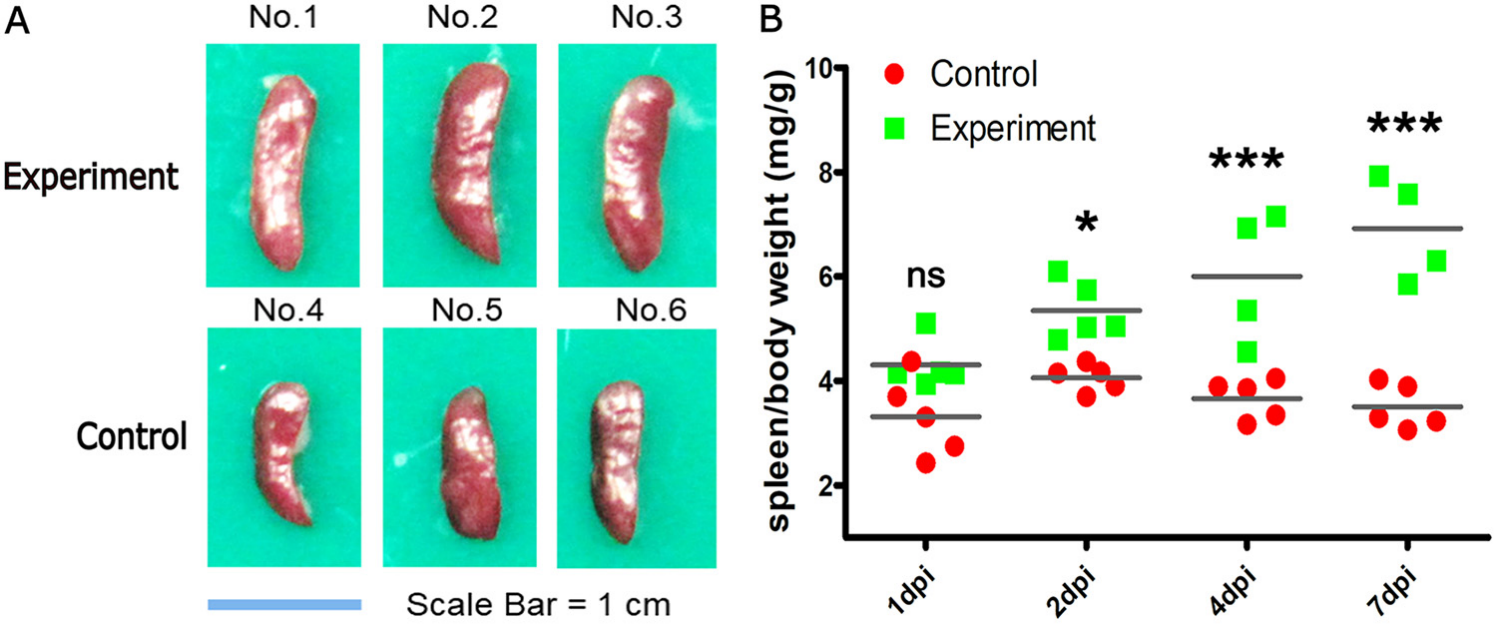
Pathological observations of the spleen from S. suis*-*infected and control mice. (A) For comparison, spleen from S. suis-infected and control mice were shown at 4 dpi. (B) The data used for statistical analysis represent the mean ± SEM of spleen/body weight (milligram per gram) from five/four mice at 1 dpi, 2 dpi, 4 dpi, and 7 dpi. *, *P < *0.05; ***, *P < *0.001; n.s., not significant compared with control (two-way ANOVA).

To explore the mechanism of splenic lesions infected by S. suis, female 6-week-old C57BL/6 mice were inoculated with S. suis serotype 2 700794 strain (5 × 10^7^ CFU), and the main gross lesions of spleen in infected mice were recorded at 1, 2, 4, and 7 days postinoculation (dpi), and the degree of splenic swelling was evaluated using the average ratio of spleen to body weight (milligrams per gram) during necropsy. The spleens of infected mice began to appear bigger from 2 dpi, with the spleen/body weight ratios of 5.346 (*P < *0.05), and showed enlargement in size relative to control spleen on 4 dpi (Fig. 1A), and the ratios increased to 6.234 (*P < *0.001) and 7.030 (*P < *0.001) at 4 dpi and 7 dpi (Fig. 1B), respectively, which is significantly different from that of control mice at the same time point.

**FIG 2:**
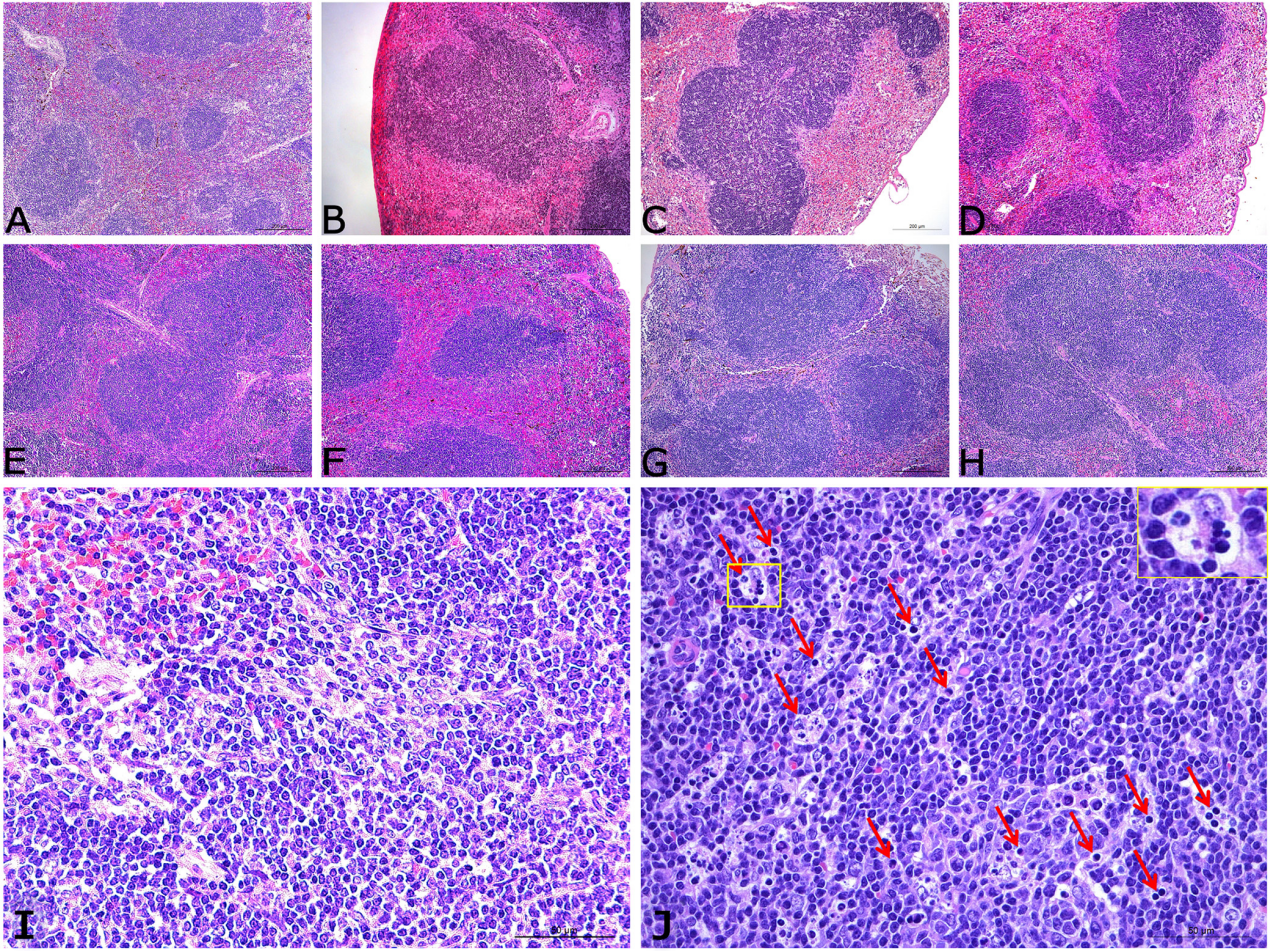
Histopathological lesions in the spleens of mice infected by S. suis. Mice were infected with 5 × 10^7^ CFU S. suis 700794, and spleen samples were collected at determined time points postinfection for histopathological evaluation. (A) Spleen from control mice. (B) Spleen at 12 hpi, showing white pulp slightly enlarged accompanied with mild lymphocyte proliferation. There was increased accumulation of red blood cells in red pulp. (C and D) Decrease in lymphocyte numbers accompanied by apoptosis in the white pulp at 1 dpi (C) and 2 dpi (D). (E) Slight decrease in lymphocyte numbers in the white pulp on 4 dpi. (F to H) There were no obvious pathological changes at 7 dpi (F), 10 dpi (G), and 14 dpi (H). Scale bars represent 200 μm (100×). (I and J) Spleen from uninfected control mice (I) compared to spleen at 1 dpi (J), showing an obvious decrease in lymphocyte numbers, apoptotic bodies (red arrows), and macrophages swallowing apoptotic cells (yellow rectangle). Scale bars represent 50 μm (400×).

The main histopathological lesions observed in the spleen of infected mice were light expansion of white pulp and slight proliferation of lymphocytes at 12 hours postinoculation (hpi) (Fig. 2B). Afterward, a decrease in lymphocyte numbers accompanied by an apoptotic characteristic in the white pulp, as well as the presence of apoptotic bodies (some of them undergoing phagocytosis) could be observed at 1 dpi (Fig. 2J), splenocyte depletion lead to the foci appeared in the white pulp at 1 dpi (Fig. 2C), and this phenomenon continued until 4 dpi (Fig. 2D and E). A small amount of macrophage infiltrating the white pulp with hemosiderin was observed at 7 dpi (Fig. 2F). No significant changes in lymphocytes, but a small amount of hemosiderin deposition, were visible in the red pulp at 10 dpi (Fig. 2G). The infected mouse spleen returned to normal, and there was no apparent pathological damage from 14 dpi (Fig. 2H). Also, from 12 hpi to 7 dpi, macrophages increased and then decreased in spleens, and phagocytizing apoptotic cells were seen clearly at 1 dpi (Fig. 2J).

### Caspase-dependent apoptosis contributes to the splenocyte depletion of infected mice by S. suis.

**FIG 3:**
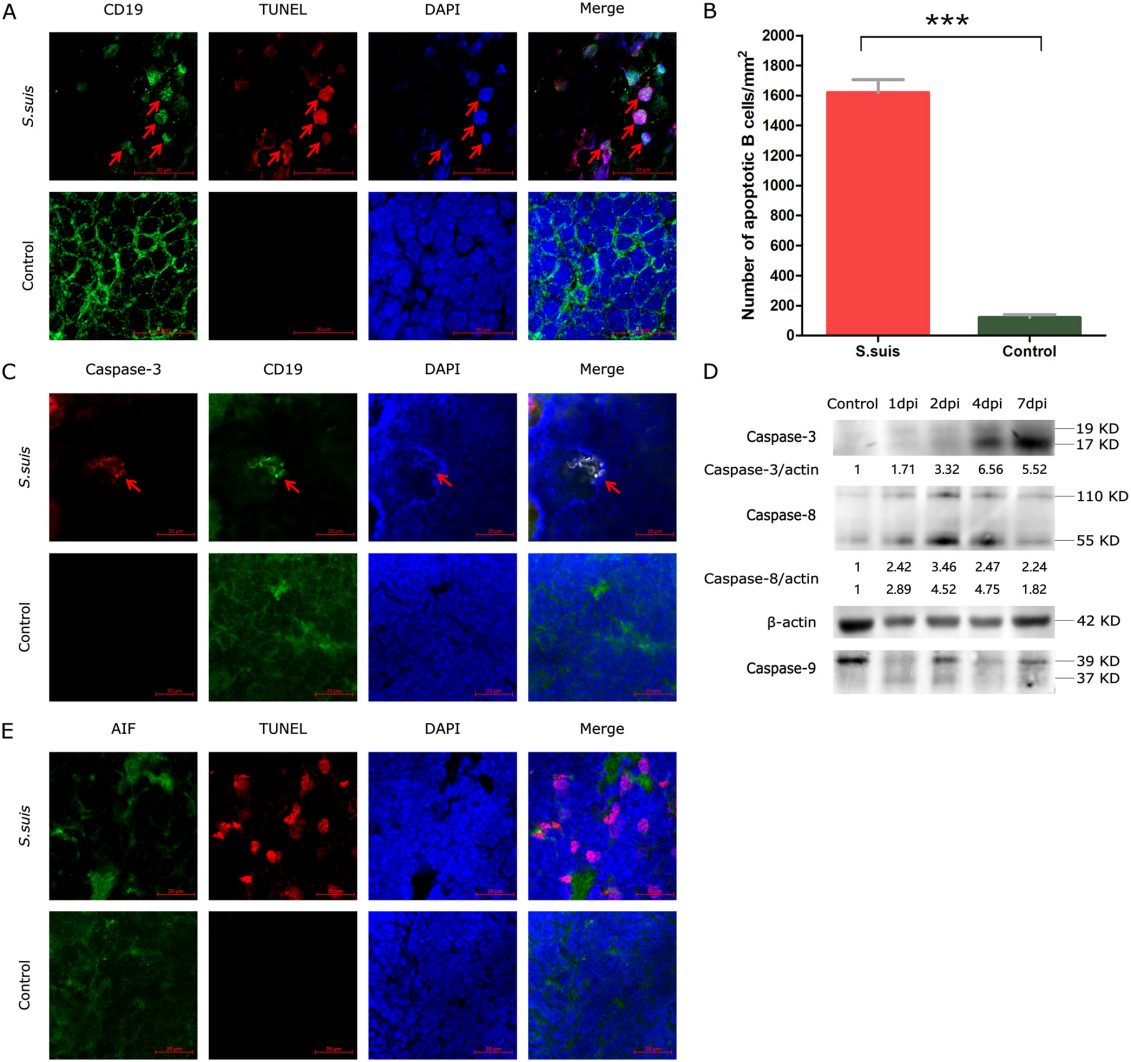
Identification of apoptotic cells and apoptosis pathway in the spleens of infected mice. (A) Appropriate FITC-conjugated antibodies were used to label CD19^+^ cells. Apoptotic cells (red) were detected by TUNEL assay, and nuclei (blue) were stained with 4′,6-diamidino-2-phenylindole (DAPI); scale bars represent 20 μm. (B) Number of apoptotic B cells in spleens from infected and control groups of mice. (C) Using confocal laser scanning microscopy, cells were stained for caspase-3 antibody (red). B cells were stained for CD19^+^ antibody (green), and nuclei were stained by DAPI (blue); scale bars represent 20 μm. (D) Levels of caspase-3, caspase-8, and caspase-9 proteins in the spleens of mice at 1, 2, 4, and 7 dpi. Spleen tissues were lysed, and Western blotting was performed. (E) Using confocal laser scanning microscopy, cells were stained for AIF antibody (green). Apoptotic cells (red) were detected by TUNEL assay, and cell nuclei were stained by DAPI (blue); scale bars represent 20 μm.

We further detected splenocyte apoptosis in the spleen lesions of S. suis-infected mice by confocal microscopy. Apoptosis was detected by using a terminal deoxynucleotidyltransferase-mediated dUTP-biotin nick end labeling (TUNEL) assay, meanwhile, to identify the type of cells that were undergoing apoptosis in the spleen; CD3^+^ T cells and B cells were labeled using fluorescein isothiocyanate (FITC)-conjugated rabbit anti-mouse CD3^+^ and CD19^+^ antibodies, respectively. The results showed that no apoptotic cells were found on CD3^+^ T cells in spleens of infected mice (data not shown), while most apoptotic signals colocalized with CD19^+^-staining B cells in sections of infected mouse spleens at 1 dpi (Fig. 3A), and the number of apoptotic B cells in the infected spleen increased significantly compared with the control spleen (Fig. 3B).

To explore which signaling pathways participate in S. suis-induced B cell apoptosis in the spleens of infected mice, proteins related to caspase and p53 pathways were detected by confocal immunofluorescence assay or Western blot analysis. Antibodies against caspase-3 related to caspase-dependent pathways were first used to label the apoptotic cells together with FITC-conjugated rabbit anti-mouse CD19^+^ antibodies. The results showed caspase-3 expression in apoptotic B cells by confocal microscopy (Fig. 3C). Western blot analysis further confirmed that S. suis induced an increase of cleavage of caspase-3. Meanwhile, the initiators caspase-8 (Fas/tumor necrosis factor [TNF] mediated) and caspase-9 (mitochondrion mediated) and CytC were also evaluated. These results revealed that caspase-8 was shown to be activated for dimerization (110-kD band) expression except for the 55-kD band in infected spleen (Fig. 3D), with significant upregulation from 2 to 4 dpi, indicating stronger signals of death receptor pathways. No significant expression change of caspase-9 and CytC indicated no involvement and enhancement of the mitochondria-mediated caspase pathways. Taken together, these results demonstrated the involvement of the caspase-dependent death receptor pathway in S. suis-induced splenic cell apoptosis.

Nuclear translocation of apoptosis-inducing factor (AIF) from mitochondria triggers chromatin condensation and DNA fragmentation to induce apoptosis (16) via the p53 pathway. To verify whether splenocyte apoptosis was induced via the p53 pathway in infected spleen, the expression of AIF was detected in apoptotic cells by immunofluorescence staining. The results showed no nuclear localization of AIF signal in apoptotic cells from infected spleen (Fig. 3E), and Western blot analysis also further showed lack of activation of AIF (data not shown). The expression of p53 analyzed by Western blotting also showed that no p53 was activated in the spleen of infected mice (data not shown). These results suggest no involvement and enhancement of the p53 pathway. Therefore, the cumulative results demonstrated that in the spleens of infected mice, the caspase pathway contributed to S. suis-induced splenic cell apoptosis.

### S. suis infection induces an M1 macrophage polarization in infected spleen.

**FIG 4:**
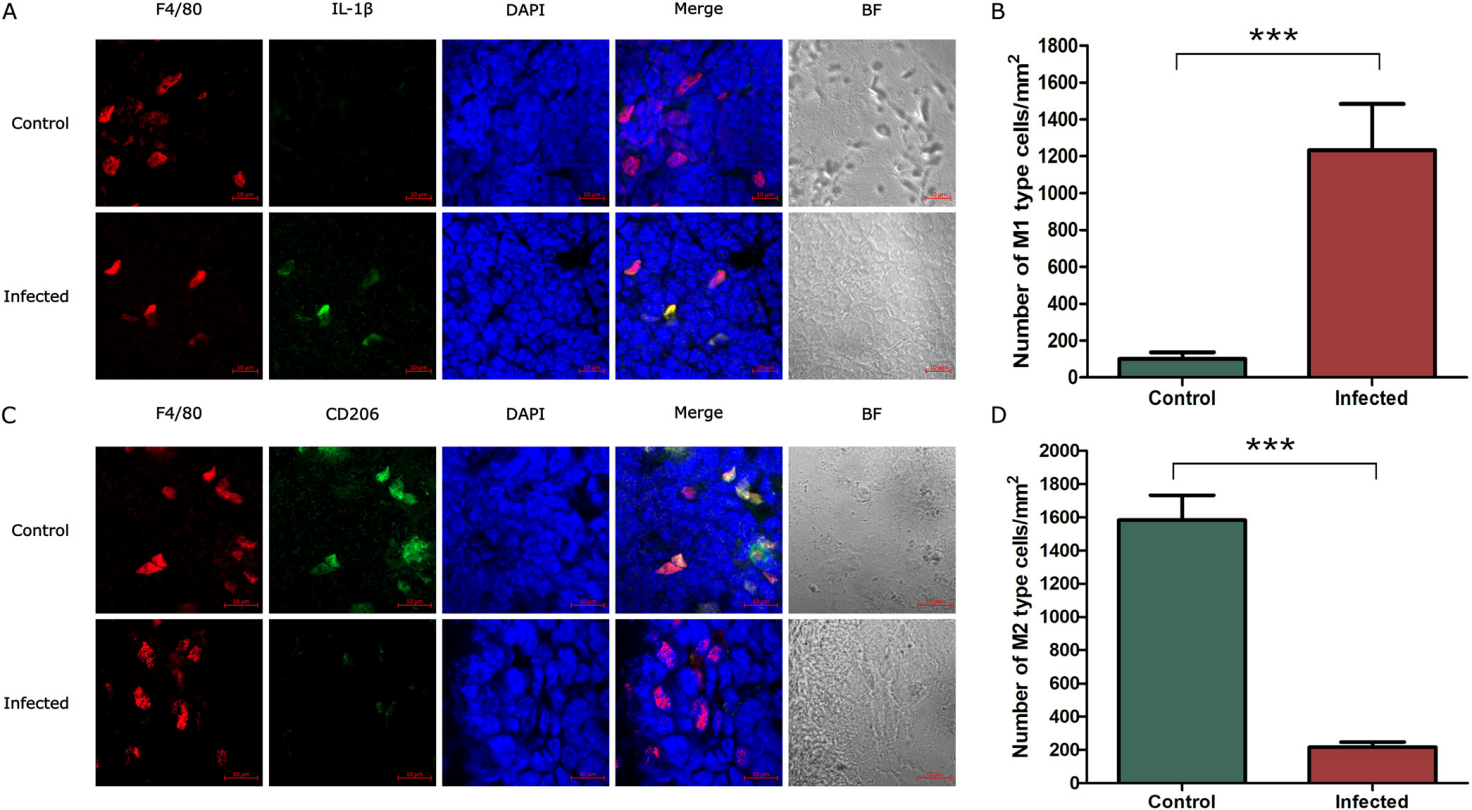
Splenic macrophage polarization in S. suis infection. Using confocal laser scanning microscopy, macrophages were stained for F4/80 antibody (red). M1 macrophages (green) were stained for IL-1β antibody (A) and the number of M1 type macrophages in spleens from infected and control groups of mice (B). M2 macrophages (green) were stained for CD206 antibody (C) and the number of M2 type macrophages in spleens from infected and control groups of mice (D). Cell nuclei were stained by DAPI (blue); scale bars represent 20 μm.

Macrophages are divided into 2 subtypes (M1 and M2) according to their activation status. M1 macrophages have proinflammatory effects and secrete proinflammatory cytokines, e.g., interleukin 6 (IL-6), tumor necrosis factor alpha (TNF-α), and IL-17A, while M2 macrophages have anti-inflammatory effects and do not secrete proinflammatory cytokines (17). To assess the type of activated macrophages in spleens of infected mice, macrophages were labeled using Alexa Fluor 647 rabbit anti-mouse F4/80 antibody, and M1 macrophages and M2 macrophages were labeled using rabbit anti-mouse IL-1β and CD206 antibodies, respectively. As shown in Fig. 4, the results showed that most confocal signals colocalized with IL-1β-labeled M1 macrophages in infected spleens (Fig. 4A), and the number of M1-type macrophages in the infected spleens increased significantly compared with the control spleen (Fig. 4B), but most confocal signals colocalized with CD206-labeled M2 macrophages in the control spleen (Fig. 4C), and the number of M2-type macrophages in the infected spleens decreased significantly compared with the control spleen (Fig. 4D). These results demonstrated that macrophages in spleen were polarized, with M2 macrophages polarized to classical M1 macrophages during S. suis infection, suggesting proinflammatory cytokines secreted by M1 macrophages may contribute to S. suis-induced splenocyte depletion in the infected- mice.

### S. suis infection actives the NLRP3 inflammasome and MAPK signaling pathways in mouse spleen.

**FIG 5:**
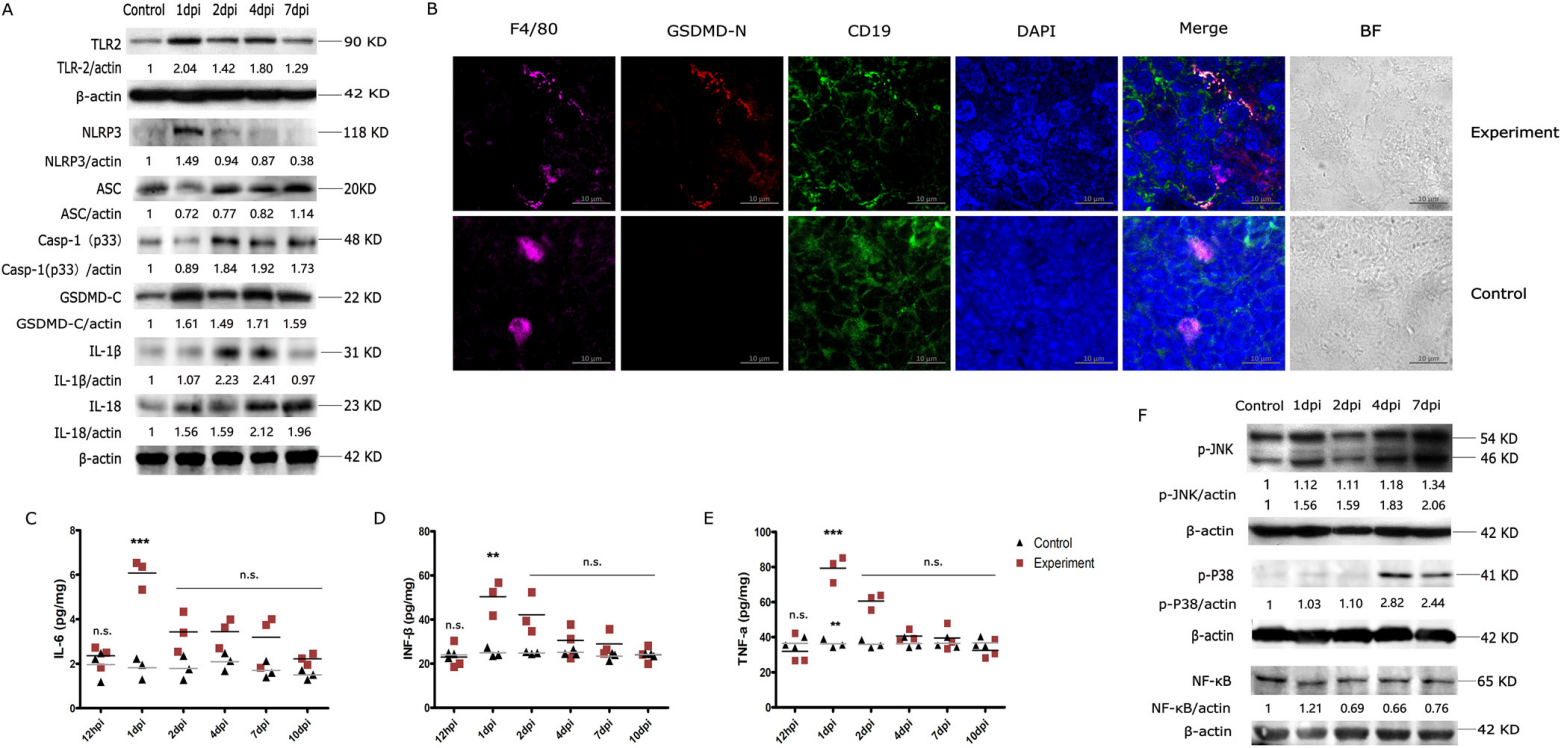
Activation of inflammation signaling pathway and identification of pyrolyzed cells in mouse spleen by S. suis infection. (A) Levels of TLR2, NLRP3, ASC, caspase-1, GSDMD-C, IL-1β, and IL-18 in the mouse spleen at 1, 2, 4, and 7 dpi. Spleen tissues were lysed, and Western blotting was performed. (B) Identification of pyrolyzed cells in mouse spleen by confocal microscopy. Macrophages were stained for F4/80 antibody (purple) and GSDMD-N antibody (red). B cells were stained for CD19^+^ antibody (green), and nuclei were stained by DAPI (blue); scale bars represent 20 μm. ELISA kits were used to detect levels (picograms per milliliter) of IL-6 (C), IFN-β (D), and TNF-α (E) at 12 hpi, 1, 2, 4, 7, and 10 dpi in spleens. The red squares represent cytokine levels of infected mice, and the black triangles represent cytokine levels of control mice. (F) Levels of p-JNK, p-p38, and NF-κB proteins in the spleen of mice at 1, 2, 4, and 7 dpi. Results are expressed as means ± SD, and significance was determined using one-way ANOVA and the Tukey multiple-comparison test. ***, *P < *0.001; **, *P < *0.01; n.s., not significant.

Inflammatory factors often contribute to tissue lesions during infectious pathogenesis. To understand the mechanisms underlying spleen lesion development and to identify the type of inflammasome that is activated during S. suis infection, we detected NLRP1, NLRP3 and AIM2 inflammasome complexes in spleens of infected mice. As shown in Fig. 5A, S. suis significantly induced the expression of Toll-like receptor 2 (TLR2) and NLRP3 at 1 dpi, whereas the expression of AIM2 did not significantly increase, and TLR4 and NLRP1 were not expressed (data not shown). Meanwhile, S. suis could induce the cleavage of pro-caspase-1 and gasdermin D (GSDMD) and increase the expression of IL-1β and IL-18 in spleens of infected mice, which suggests that S. suis caused pyroptosis in splenocytes. Furthermore, confocal microscopy showed that pyrolyzed cells were macrophages (Fig. 5B). Correspondingly, the levels of proinflammatory cytokines IL-6, IFN-β, and TNF-α reached a peak at 1 dpi (6.08 pg/mL, 50.33 pg/mL, and 79.35 pg/mL, respectively) and then began to decrease in infected spleens (Fig. 5C to E). These results indicated that the NLRP3 inflammasome-related pathway was activated and induced proinflammatory immune responses in mouse spleens during S. suis infection. MAPKs are classical inflammation-related signaling molecules that include JNK, ERK1/2, and p38, and they are involved in regulating cellular responses to cytokine stimulation (18). In this study, total phosphorylated JNK, ERK1/2, and p38 levels were detected by Western blotting, and the results showed that S. suis upregulated JNK and p38 phosphorylation in infected spleens compared with the control group (Fig. 5F), but there were no changes in ERK1/2 phosphorylation (data not shown), which indicated that S. suis regulated the inflammatory response in spleens of mice by activating the JNK and the p38 pathways, but not the ERK pathway. Also, the NF-κB protein had a slight increase at 1 dpi and decreased from 2 dpi to 7 dpi. Taken together, NLRP3 inflammasome and MAPK signaling pathways are involved in proinflammatory responses in spleens of infected mice.

### S. suis infection inhibits anti-inflammatory responses in spleen.

**FIG 6:**
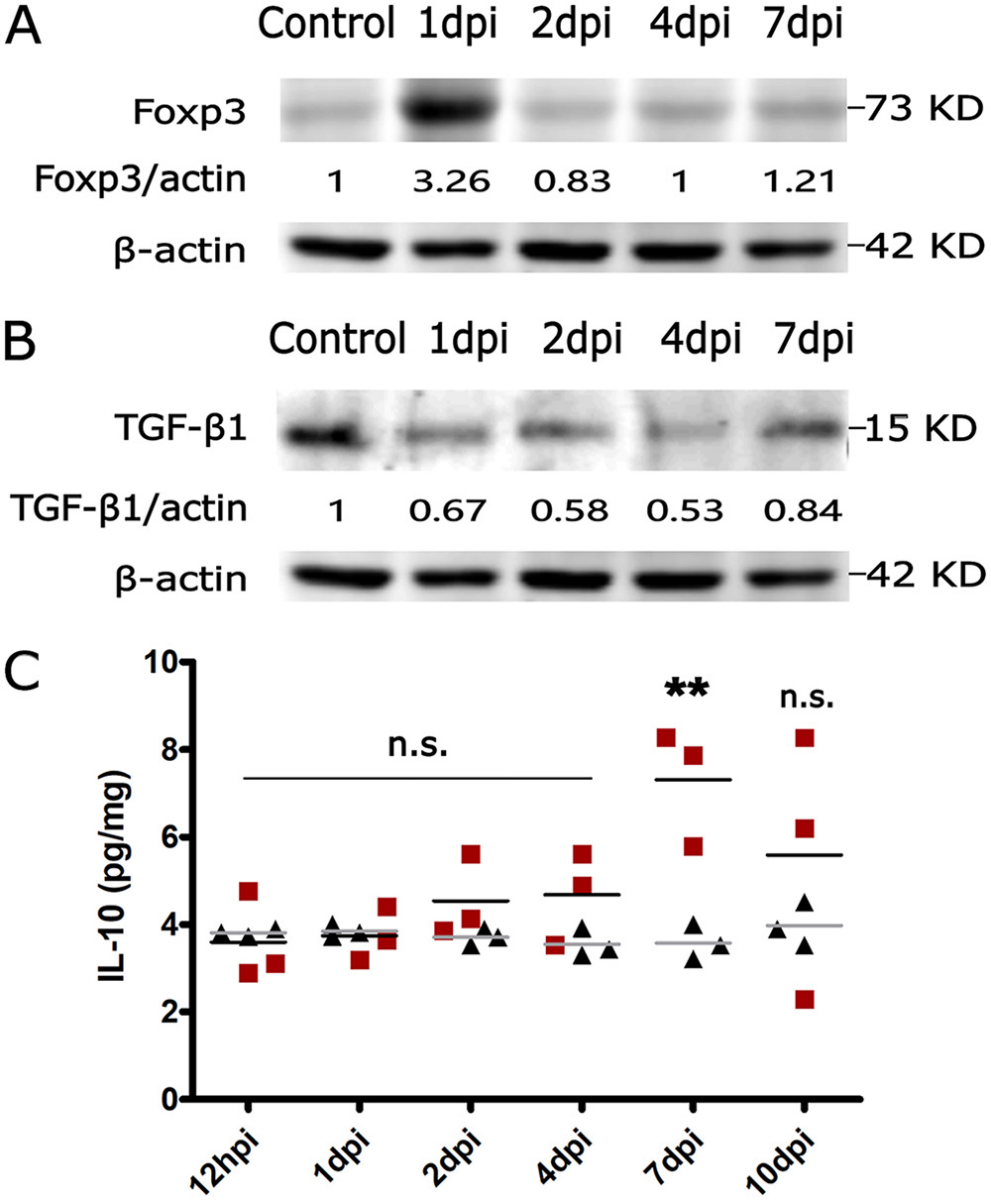
S. suis infection inhibited anti-inflammatory immune responses in infected mouse spleen. (A) Level of Foxp3 protein in the spleen of control and infected mice at 1, 2, 4, and 7 dpi. Spleen tissues were lysed, and Western blotting was performed. (B) Levels of TGF-β1 protein in the spleen of control and infected mice at 1, 2, 4, and 7 dpi. Spleen tissues were lysed, and Western blotting was performed. (C) ELISA kits were used to detect levels (picograms per milliliter) of IL-10 at 12 hpi, 1, 2, 4, 7, and 10 dpi in the spleen. The red squares represent cytokine levels of infected mice, and the black triangles represent cytokine levels of control mice. Results are expressed as means ± SD, and significance was determined using one-way ANOVA and the Tukey multiple-comparison test. **, *P < *0.01; n.s., not significant.

Regulatory T cells (Tregs) play an important role in the regulation of T cell-mediated immune responses through suppression of T-cell proliferation and secretion of inhibitory cytokines, such as transforming growth factor β1 (TGF-β1) and IL-10 (19). First, we detected CD25^+^ Foxp3^+^ Treg cell development by detecting the expression of the Foxp3 protein. The result showed that S. suis significantly increased the development of Treg cells at 1 dpi of early infection (Fig. 6A), which suggests that T cell proliferation and secretion of inhibitory cytokines were suppressed. Correspondingly, we investigated the anti-inflammatory cytokines with antagonistic effects, including TGF-β1 and IL-10. The results showed decreased secretion of TGF-β1 from 1 to 7 dpi in infected spleens (Fig. 6B), and the level of IL-10 had no significant increase from 12 hpi to 4 dpi but increased significantly only at 7 dpi (47.31 pg/mL) in spleens from infected mice compared with control ones (Fig. 6C). TGF-β1 and IL-10 negatively regulate the inflammatory response and reduce the activity of the proinflammatory factors (20). Thus, these data suggest that anti-inflammatory responses in spleen may be inhibited at the early stage of S. suis infection.

### Splenocyte depletion induced by S. suis alleviated in TNF-α^−/−^ mice.

**FIG 7:**
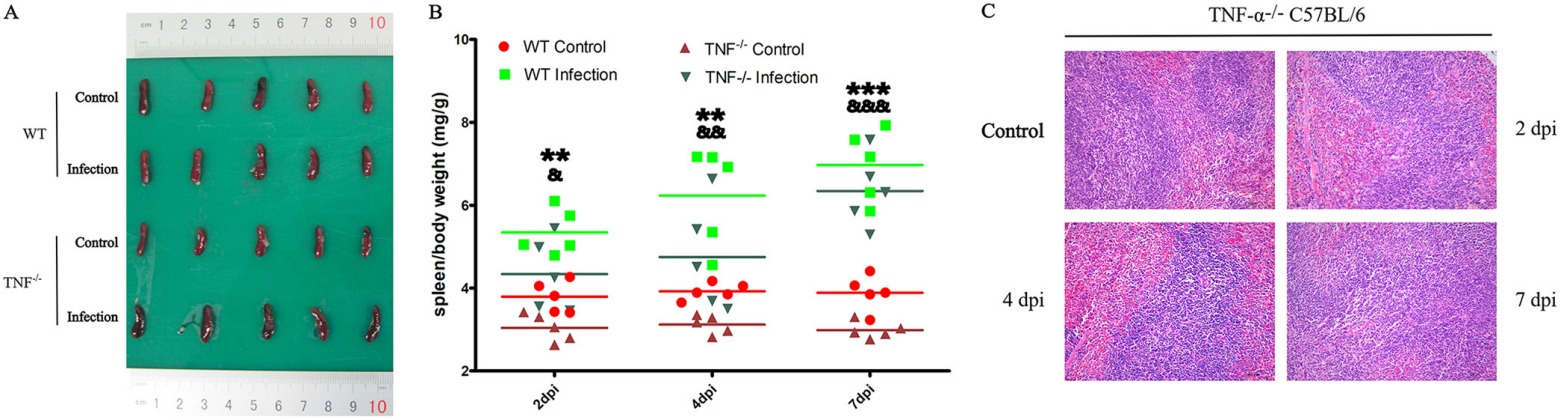
Pathological observations of the spleen from TNF-α^−/−^ mice. (A) For comparison, spleens from WT mice and TNF-α^−/−^ mice are shown at 2 dpi. (B) The data used for statistical analysis represent the mean ± SEM of spleen/body weight (mg/g) from five mice at 2 dpi, 4 dpi, and 7 dpi. (C) Spleens from TNF-α^−/−^ mice at 2, 4, and 7 dpi compared to spleen from TNF-α^−/−^ control mice, showing no significant change in the number of lymphocytes at 2 and 4 dpi and a slight decrease in the number of lymphocyte at 7 dpi. Scale bars represent 200 μm (100×).

TNF-α was significantly upregulated at 1 dpi, and caspase-8 was significantly upregulated from 1 to 4 dpi, which indicated that the TNF signaling pathway may be activated. To investigate the role of TNF-α in S. suis-induced splenocyte depletion, we used S. suis 700794 to infect TNF-α^−/−^ mice. As shown in Fig. 7, it was found that the number of spleen lymphocytes of the infected TNF-α^−/−^ mice had no obvious change at 2 and 4 dpi and slightly reduced only at 7 dpi. S. suis-induced splenocyte depletion is alleviated in TNF-α^−/−^ mice in the early stage of infection compared with the results in Fig. 2. The results indicate that TNF-α played an important role in the splenocyte depletion induced by S. suis.

## DISCUSSION

S. suis serotype 2 is considered an emerging pathogen and represents a threat to the health of both humans and animals (1, 4). The pathogenic mechanisms have been widely explored; however, the immune response mechanisms caused by S. suis serotype 2 are largely unknown. In this study, we found S. suis serotype 2 infection could cause damage to the spleen. The spleen is an important peripheral immune organ where immune cells gather and immune response occurs (21, 22). It plays an important role in the immune response by detecting pathogens and producing white blood cells in response (23). Here, we have found that S. suis infection results in splenic edema with splenocyte depletion in mice, and there are fluids in the interstitial spaces of the infected spleen. Marked pathological changes of the spleen, characterized by splenomegaly and a decrease in lymphocyte numbers, suggest that S. suis infection likely modulates host immunity.

**FIG 8:**
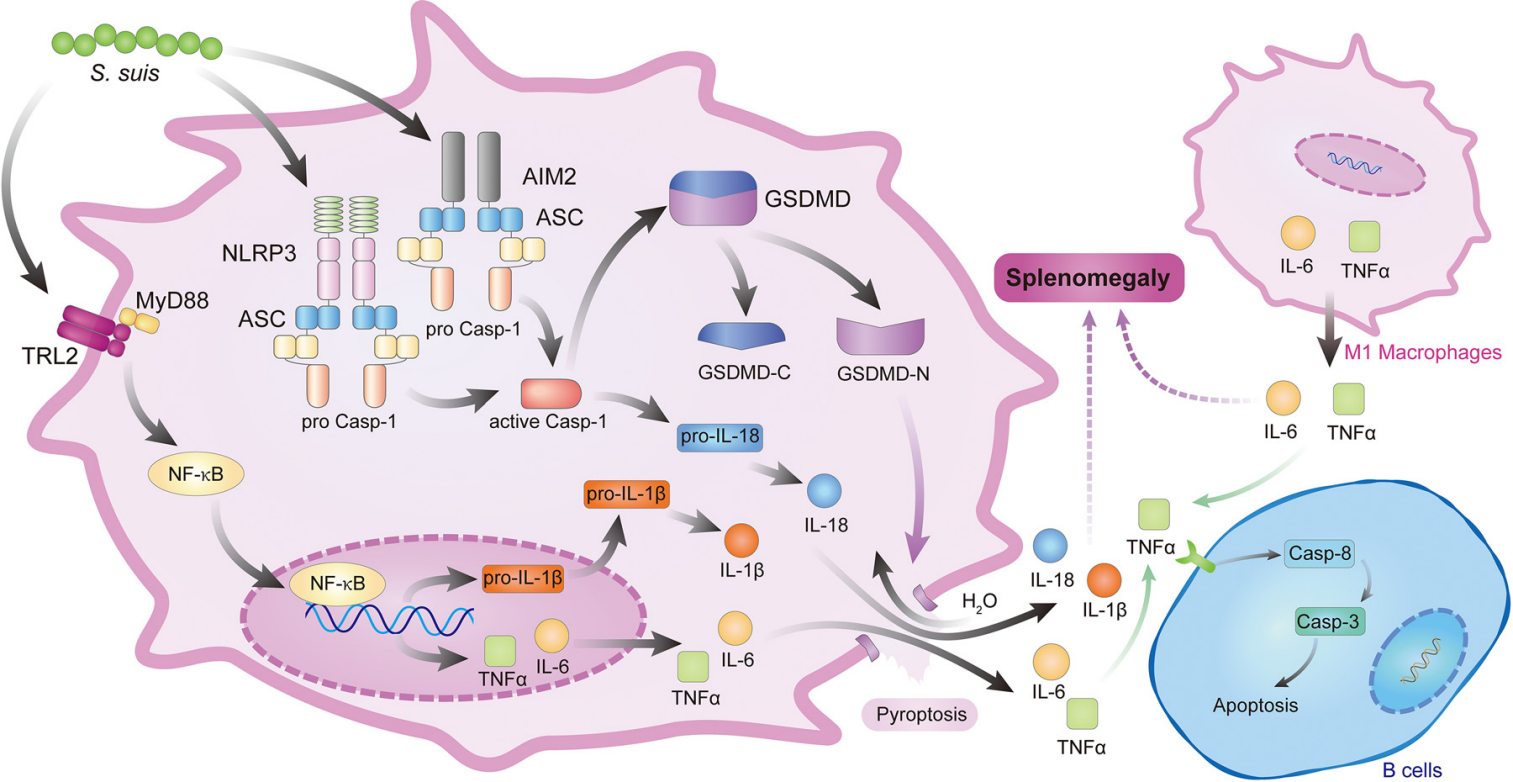
S. suis infection causes splenomegaly through the polarization and pyroptosis of M1 macrophages and inflammation through inflammasomes and MAPK signaling pathways, and it induces apoptosis of splenic B cells through the caspase-dependent signaling pathway. S. suis reaches the spleens and leads to the polarization of the spleen macrophages toward M1 type and pyroptosis. Macrophage pyroptosis is via the inflammasome/caspase-1/GSDMD signaling pathway, which promotes the release of mature IL-18 and IL-1β. Then, mature IL-18 and IL-1β promote the release of other proinflammatory cytokines. TNF-α increases and induces B cells apoptosis via the TNF receptor pathway.

Apoptosis is important for development and tissue homeostasis (24, 25). Pathogens induced modulation of host cell death pathways that may help eliminate key immune cells or evade host defenses that limit the infection (26, 27). In this study, S. suis-induced splenic lymphocyte disruption was accompanied by apoptosis in mice, suggesting that apoptosis of splenic B cells is very likely the direct cause of this disruption and affects immunological functions and disease resistance. We found, by TNF-α^−/−^ mouse infection assay, that TNF-α plays an important role in the depletion of splenocytes. TNF-α was released through activation and pyroptosis of M1 macrophages, which may target B cells to cause apoptosis through the TNF receptor (Fig. 8). There are several pathways that can induce apoptosis (28, 29). Here, our results showed that S. suis triggers caspase-dependent and AIF-independent apoptosis; meanwhile, detection of multiple proteins verified that the activation of caspase-3 is only through the death receptor pathways. Taken together, these results indicate that the apoptosis signaling pathway in the spleen is different from the thymus (7), although S. suis triggers apoptosis in the same infection model.

Splenomegaly is a consequence of lesions and an important clinical indicator of S. suis infection. However, knowledge of the mechanisms underlying spleen lesions that are S. suis induced is still very limited. Inflammatory responses play an important role in pathological damages such as hepatic fibrosis, splenic fibrosis, and kidney inflammation during microbial infection (30, 31). Macrophages are important cells of innate immunity (32, 33), whose functions are settled in response to microenvironmental signals, which drive the acquisition of polarization programs (34–36). In our study, the macrophages are first polarized to proinflammatory M1 phenotype and secrete proinflammatory cytokines to assist the host against pathogens and become the main splenic macrophage when macrophages receive S. suis signals from the surrounding microenvironment in the acute infection phase. Meanwhile, we found that the activation of NLRP3 inflammasome cleaved procaspase-1 to caspase-1, which cleaved pro-IL-1β and pro-IL-18 into mature IL-1β and IL-18. GSDMD in M1 macrophages was cleaved by caspase-1 to generate GSDMD-N fragments that form large transmembrane pores, allowing the release of IL and TNF-α and driving pyroptosis, which makes a large number of proinflammatory cytokines, including IL-1, IL-17A, and TNF-α to be released subsequently (Fig. 8), which may be able to chemoattract neutrophils, T cells, and eosinophils to migrate to the spleen tissues. Meanwhile, classic inflammation-related JNK and p38 MAPK signaling pathways were activated, which are also involved in proinflammatory responses. In addition, the JNK-modulating inflammasome signaling pathway might be a common mechanism in host-pathogen interactions (37). The relationship between NLRP3 inflammasome activation and phosphorylation of JNK in macrophages during S. suis infection will be further verified in future studies.

Splenic lymphocytes play a crucial role in the immune system (38, 39), and Treg cells are a specialized subpopulation of T cells that act to suppress the anti-inflammatory response, thereby maintaining homeostasis and self-tolerance (40). We found that the Treg cell expression was upregulated significantly at 1 dpi by detecting Foxp3 protein, which indicates that Treg cells may inhibit anti-inflammatory cytokine, such as TGF-β1 and IL-10, production in spleen during S. suis infection. Indeed, the expression of regulatory cytokine IL-10 was not significantly upregulated until 7 dpi, and production of TGF-β1 also was inhibited by detecting TGF-β1-downregulated expression, which has been recognized as an important inhibitory cytokine in the immune system. These data demonstrate that the production of inhibitory cytokines is blocked, suggesting that anti-inflammatory immune responses are suppressed in the early infection. Collectively, proinflammatory immune response dominates in the early stages of infection. As a result, the release of inflammatory mediators increases vascular permeability and damages the trabecular structure of the spleen. Then, liquid components and red blood cells in the blood penetrate into the interstitial spaces, which causes splenomegaly and a large number of red blood cells in red pulp and white pulp. In addition, proinflammatory cytokines also induce splenocyte apoptosis (Fig. 8).

In conclusion, we found, for the first time, that S. suis infection induced NLRP3 inflammasome activation, macrophage M1 polarization, and B cell apoptosis in infected spleens. Moreover, it is also the first report that NLRP3 inflammasome and MAPK signaling pathways are involved in inflammation, which contributes to spleen damage caused by S. suis. Induction of splenic damage may lead to host immune depression and appears to be a new virulence mechanism of S. suis.

## MATERIALS AND METHODS

### Animal ethics statements.

This study was carried out in accordance with recommendations from the Guide for the Care and Use of Laboratory Animals of the Ministry of Science and Technology of China. The protocols were reviewed and approved by the Committee on the Ethics of Animal Experiments of the Harbin Veterinary Research Institute of the Chinese Academy of Agricultural Sciences. Mouse challenge experiments (approval number SY-2018-MI-012) and TNF-α^−/−^ C57BL/6 mouse challenge experiments (approval number SY-2020-MI-009) with S. suis were conducted within the animal biosafety level 2 facilities in the Harbin Veterinary Research Institute of the Chinese Academy of Agricultural Sciences (CAAS).

### Bacterial strain and animal inoculation.

According to our previous studies (7), briefly, 35 female 6-week-old C57BL/6 mice were inoculated intraperitoneally (i.p.) with S. suis serotype 2 700794 strain (5 × 10^7^ CFU). In parallel, and as controls, 35 female 6-week-old C57BL/6 mice were inoculated with sterile Todd-Hewitt broth (THB). Five infected mice and five control mice were humanely euthanized at 12 h postinoculation (hpi) and 1, 2, 4, 7, 10, and 14 days postinoculation (dpi). Spleen samples were dissected from each mouse, and then splenic lesions were evaluated as spleen/body weight (milligrams per gram) ratios. Moreover, the TNF-α^−/−^ mice of the C57BL/6 background were purchased from Cyagen Biosciences (Santa Clara, USA), and 30 (15 male, 15 female) TNF-α^−/−^ mice at 7 weeks were randomly divided into 6 groups (*n* = 5 of each group). In parallel, and as controls, 30 (15 male, 15 female) 7-week-old C57BL/6 mice were randomly divided into 6 groups. TNF-α^−/−^ control mice, C57BL/6 control mice, TNF-α^−/−^-infected mice, and C57BL/6-infected mice were inoculated intraperitoneally with 0.2 mL THB or 0.2 mL S. suis 700794 (2 × 10^7^ CFU), respectively. The samples from spleen were harvested at 2, 4, and 7 dpi.

### Histopathology examinations.

Samples of the spleen of mice were fixed in 4% neutral buffered formalin solution, then embedded in paraffin, cut into 6- to 8-μm-thick slices, and stained with hematoxylin and eosin (H&E). Spleen lesions were observed in a blinded manner under an upright fluorescence microscope, DM4000B (Leica, Frankfurt, Germany).

### Confocal microscopy.

Mouse spleen samples were sectioned (7-μm-thick slices) on a cryostat and used for double-immunofluorescence staining. Apoptosis was detected using a terminal deoxynucleotidyltransferase-mediated dUTP-biotin nick end labeling (TUNEL) assay by the *in situ* cell death detection kit (Roche, Mannheim, Germany). To confirm the types of cells that underwent apoptosis or pyroptosis, spleen sections were stained with different antibodies, including FITC-conjugated rabbit anti-mouse CD3, CD19 antibody (1:50, Southern Biotech), Alexa Fluor 647 rabbit anti-mouse F4/80 antibody (1:50; Abcam, Cambridge, UK), rabbit anti-mouse caspase-3, gasdermin D-N (GSDMD-N) antibody (1:300; Cell Signaling, Danvers, MA, USA), and Alexa Fluor 568-conjugated goat anti-rabbit antibody (1:500; Sigma, Saint Louis, MO, USA). To confirm that apoptosis-inducing factor (AIF) translocated to the nucleus, spleen sections were stained with rabbit anti-mouse AIF monoclonal antibody (1:50; Abcam, Cambridge, UK) and Alexa Fluor 488-conjugated goat anti-rabbit antibody (1:1,000; Sigma). To confirm the type of activated macrophages, spleen sections were stained with Alexa Fluor 647 rabbit anti-mouse F4/80 monoclonal antibody (1:50; Abcam, Cambridge, UK), rabbit anti-mouse interleukin-1β (IL-1β), or CD206 polyclonal antibody (1:50; ABclonal, Beijing, China) and Alexa Fluor 488-conjugated goat anti-rabbit antibody (1:1,000; Sigma). Finally, nuclei were stained with 4′,6-diamidino-2-phenylindole (DAPI; Sigma). Sections were observed by a laser-scanning confocal microscope. For the morphometric analysis, the average number of TUNEL-positive B cells and M1/M2 polarization in the spleen were counted in 10 fields under a confocal microscope. The number of labeled cells per unit area (millimeters squared) was calculated, and the result was expressed as mean values.

### Western blotting.

Spleen tissues were incubated on ice with cell lysis buffer containing a protease inhibitor cocktail (catalog no. 04693132001; Roche, Bern, Switzerland) and 0.1 mM phenylmethylsulfonyl fluoride (PMSF) for 2 h. Cell lysates were centrifuged at 12,000 × *g* for 30 min at 4°C, and protein concentration was determined by the bicinchoninic acid (BCA) protein assay kit (Beyotime, Shanghai, China). Equal amounts of protein were loaded in 12% (wt/vol) SDS-PAGE gels (Boster Biological Technology, Suzhou, China) and transferred to polyvinylidene fluoride (PVDF) membranes (MerckMillipore, Darmstadt, Germany). After blocking with 10% dry milk at 4°C overnight, the membranes were incubated for 1 h at room temperature with different antibodies, including anti-caspase-3, anti-caspase-8, anti-caspase-9, anti-p53, anti-CytC, anti-AIF, anti-p-JNK, anti-p-p38, anti-NLRP3, anti-NLRP1, anti-AIM2, anti-caspase-1, anti-IL-1β, anti-IL-18, anti-GSDMD-C, anti-Foxp3, anti-transforming growth factor β1 (TGF-β1; 1:1,000; Abcam), and anti-β-actin (1:200,000; ABclonal). After washing, the membranes were incubated with Dylight 800 labeled goat anti-rabbit IgG (SeraCare Life Sciences, Milford, MA, USA) for 1 h, and immunoreactive bands were visualized using the near-infrared fluorescence scanning imaging system (Odyssey CLx, Licor, America).

### Cytokine analysis.

One hundred milligram mouse spleen tissues collected at 12 hpi and 1, 2, 4, 7, and 10 dpi were rinsed with PBS, homogenized in 1 mL of PBS, and stored overnight at −20°C. After two freeze-thaw cycles were performed, the homogenates were centrifuged for 5 min at 5,000 × *g* at 2 to 8°C. The supernatants were used for the detection of levels of IL-6, IL-10, interferon β (IFN-β), and tumor necrosis factor α (TNF-α) by commercial enzyme-linked immunosorbent assay (ELISA) kits (Cusabio, Hangzhou, China) according to the manufacturer’s instructions. The number of cytokines (picograms per milligram) was calculated according to a standard curve generated from standards supplied in different cytokine commercial kits.

### Statistical analysis.

Numerical data are expressed as the mean ± standard error of the mean (SEM) and were analyzed using GraphPad Prism software (version 5.0; GraphPad Software Inc.). Differences between groups were assessed using two-way analysis of variance (ANOVA) or one-way ANOVA and the Tukey multiple-comparison test. A *P* value of less than 0.05 was considered statistically significant.
